# Research Advances in the Plant–Nematode Interaction

**DOI:** 10.3390/life13081722

**Published:** 2023-08-10

**Authors:** Jia You, Yanfeng Hu, Jingsheng Chen

**Affiliations:** 1 Institute of Pratacultural Science, Heilongjiang Academy of Agricultural Science, Harbin 150086, China; rokiyou@126.com; 2 Northeast Institute of Geography and Agroecology, Chinese Academy of Sciences, Harbin 150081, China; 3 College of Biology and Food Engineering, Chongqing Three Gorges University, Chongqing 404000, China; jingsheng6673182@163.com

Nematodes, which are highly important and abundant animals in the animal kingdom, demonstrate remarkable adaptability to various environments. Among them, plant-parasitic nematodes (PPNs) pose a major threat to agricultural crops. It is crucial to timely and accurately detect the dynamics underlying PPN occurrence across different agricultural regions for the effective control of these pests. Moreover, the current reliance on chemical nematicides for PPN management in crop production raises concerns due to their harmful effects on human health and the environment. The shift towards sustainable agriculture necessitates the adoption of an integrated approach for PPN management, combining safe traditional methods, such as host–plant resistance, crop rotation, and soil amendment, alongside novel techniques, like biocontrol and genome editing. On the other hand, a comprehensive understanding of the relationship between the soil nematode communities and their environment holds promise for controlling agricultural pests and improving crop yields. This Special Issue of *Life* features seventeen articles contributed by scholars in the field of the plant–nematode interaction. Their research efforts have primarily focused on the following areas: PPN identification, the development of effective and safe control methods, disease-resistant breeding, and exploring the ecological function of soil nematode communities in agricultural systems and crop health.

Symptoms caused by most of the PPNs are largely inapparent due to their small size and their feeding on roots. Hence, PPN diseases often remain overlooked, making the accurate and prompt detection of PPNs crucial for devising effective control strategies. In a study by Pan et al. [1], a combination of morphological and molecular approaches was employed to identify an important quarantine PPN, *Ditylenchus destructor*, from the maize seedlings. This study was the first report of *D. destructor* causing stem rot of maize in Heilongjiang province, China. Mo et al. [2] performed the systematic investigation of PPNs associated with main subtropical crops in Guangxi province, China. Through analysis of 425 soil and root samples from crops, including sugarcane, rice, maize, and soybean, they identified a total of 48 order/family/genera of PPNs. The five highest frequency of PPNs were *Tylenchorhynchus*, *Pratylenchus*, *Helicotylenchus*, *Meloidogyne*, and *Hirschmanniella*. Their study also highlighted that the types of host plant was closely related to the occurrence of PPNs. Zoubi et al. [3] conducted a detailed survey about the occurrence, distribution, and diversity of PPNs in the main citrus tree-growing regions of Morocco between the years 2020 and 2021. Their results suggested that the predominant PPN species in citrus roots and soil were *Tylenchulus semipenetrans* (88%), *Helicotylenchus* spp. (75%), *Pratylenchus* spp. (47%), *Tylenchus* spp. (51%), and *Xiphinema* spp. (31%). The article by Sikandar et al. [4] documented the occurrence of PPNs with maize and other rotational crops from Punjab, Pakistan. Their study indicated that the most abundant PPNs were *Heterodera zeae* (55.71% incidence), *Tylenchorhynchus elegans* (33.33%), and *Helicotylenchus certus*. Their findings emphasized the potential threats posed by these nematode species to maize and other rotational crops in Punjab, requiring special attention from researchers. Jiang and colleagues [5] developed a sensitive and specific SCAR-PCR technique for the effective detection of the sugar beet cyst nematode (*Heterodera schachtii*) from infected plant root and soil samples. This technique served as a valuable tool for the accurate detection of *H. schachtii* and can aid in the management of this nematode species in sugar beet fields.

Currently, the most effective management of PPNs is to develop resistant cultivars in crops, while identification of novel resistant genetic sources and loci are very important for constructing cultivars with PPN resistance. In this context, a genome-wide association study (GWAS) was carried out by Sohail et al. [6] to screen single-nucleotide polymorphism (SNP) markers for root disease resistance (*Pratylenchus* and *Fusarium* species) from a panel of 198 elite spring wheat accessions. Ultimately, they identified eleven different SNPs on chromosomes 1A, 1B, 2A, 3A, 4A, 5B, and 5D, all which were significantly associated with root-lesion nematode resistance; eight different markers on chromosomes 1A, 2B, 3A, 4B, 5B, and 7D were responsible for *Fusarium* crown rot resistance. The soybean cyst nematode (SCN), *Heterodera glycines*, is a crucial thread to soybean (*Glycine max* (L.) Merr.) production worldwide, including China. To uncover potential valuable genotypes for breeding new SCN-resistant cultivars, Chen et al. [7] performed pot assays to assess the resistance of 110 domestic commercial soybeans from different regions of Northeast China to two predominant SCN populations, HG types 7 and 1.3.4.7, in a soybean-producing region of Northeast China. Twenty-four commercial cultivars were resistant or moderately resistant to HG Type 7, whereas five accessions were classified as resistant or moderately resistant to HG Type 1.3.4.7. In comparison, Kangxian 12 and Qingdou 13 demonstrated their effective resistance to the common HG types 7 and 1.3.4.7. and can be used in the SCN resistance program. Based on phenotypic screening methods, Dalamu and colleagues [8] examined the resistant genotype of 94 native potato accessions from different parts of India against the late blight and potato cyst nematode (*Globodera* spp.). They identified three promising accessions (Garlentic, Jeevan Jyoti, and JG-1) that exhibited a high level of resistance against *G. pallida* and *G. rostochiensis*; JG-1, Kanpuria Safed, and Rangpuria were significantly resistant to late blight. These authors further employed six gene-specific molecular markers (*CosA210*, *TG689141,57R452*, *Gro1-4-1602*, *Ro1,4*, *HC276*, and *GpaVvrn*) to further confirm these PCN-resistant resources containing specific published R genes. Together, the results from these studies could help to expand our knowledge of the source of resistance in germplasm and genomic regions controlling resistance against PPNs.

Considering the development of eco-friendly and sustainable PPN control interventions in the agricultural sector, the use of organic amendments and biocontrol agents have been proposed as effective and much safer alternatives for PPN management. As detailed in this Special Issue, Yan et al. [9] reported that the secondary metabolite scopoletin, produced by the fungal strain *Penicillium janthinellum*, Snef1650, exhibits a high level of nematicide activity against *H. glycines*. The field experiments they conducted further demonstrated that using scopoletin as seed coats significantly decreased the cyst density in the soybean field. This finding indicated that the application of Snef1650 is a promising biocontrol agent for *H. glycines* control. Another article focused on the in vitro nematicidal activity of cotton seed cake (CSC) extract against *M. incognita* in tomato plants [10]. Soil biodisinfestation is a process generated after the incorporation of organic amendments followed by a plastic cover to suppress soilborne diseases. The biodisinfestation practices adopted in several Egyptian strawberry farms have shown positive effects against root-knot nematodes (RKNs, *Meloidogyne* spp.) [11]. The activation of specific microbial activity and the release of other nematicidal compounds may contribute to RKN-soil suppressiveness at the strawberry farms [11]. Berger and colleagues [12] found that the inundation process of two types of tare soils (namely sedimentation basins and the Brukner basin) during sugar beet processing efficiently decreased the viability of *G. pallida* and *H. schachtii* under laboratory-controlled conditions. These findings offer promising strategies for eco-friendly PPN control in agriculture. 

Another key topic that has emerged in this Special Issue is the impact of current agricultural practices on soil nematode structure. For example, the composition, metabolic footprint, and ecological indices of soil nematodes in monocultures of pumpkin and melon were investigated by Zhao et al. [13]. They found that monocultures of pumpkin and melon altered the soil nematode community via soil properties, with factors such as total nitrogen, total phosphorus, alkaline-N, and pH identified as the main drivers underlying this change. Argyropoulou et al. [14] examined how the addition of three aromatic plants (i.e., spearmint, peppermint, and rosemary) as soil amendments in tomato seedbeds affect the community of soil nematodes. Their findings indicated that the application of all three aromatic plants into the soil led to an increase in the abundance of free-living soil nematodes, resulting in the development of vigorous and enriched soil. Interestingly, Zeng et al. [15] reported that their long-term cultivation of transgenic maize did not exert any significant effects on the soil nematode community.

In the review paper titled “Exploiting Plant–Phytonematode Interactions to Upgrade Safe and Effective Nematode Control”, Abd-Elgawad [16] provides an overview of the economic losses caused by PPNs and the common control methods that are employed against these pests and emphasized the importance of optimizing sampling and extraction methods for effective PPN control. Additionally, their review highlights more recent studies that have focused on plant–nematode interactions, with a particular emphasis on the utilization of emerging molecular techniques in optimizing control measures for PPNs. Another review article by Abd-Elgawad [17] delved deeper into the importance of the beneficial *Xenorhabdus* bacteria in the management of agricultural pests and pathogens.

We hope that this Special Issue will stretch the knowledge of the limited research on plant–nematode interactions in agroecosystems, and engage more specialists interested in this exciting field to improve control measures for PPNs.
